# Randomised controlled trial of a smartphone application‐based dietary self‐management program on haemodialysis patients

**DOI:** 10.1111/jocn.15627

**Published:** 2021-01-28

**Authors:** Songyi Pack, Jia Lee

**Affiliations:** ^1^ Graduate School Kyung Hee University Seoul Korea; ^2^ College of Nursing Science Kyung Hee University Seoul Korea

**Keywords:** diet, haemodialysis, mobile applications, patient, self‐management

## Abstract

**Aims and objectives:**

To develop a smartphone application‐based dietary self‐management program for haemodialysis patients and to examine its effects on biochemical indicators, self‐efficacy and quality of life.

**Background:**

Lack of dietary management in haemodialysis patients can lead to serious complications such as oedema, hyponatremia, hyperkalaemia, hypertension, uraemia and eventually death; however, studies using smartphone applications for dietary self‐management in haemodialysis patients are rare.

**Design:**

A prospective, single‐blind, randomised, controlled design with repeated measures was followed with 75 haemodialysis patients at a haemodialysis centre. Data were collected from 10 January 2017–6 May 2018. The study applied the Consolidated Standards of Reporting Trials statement.

**Methods:**

The 8‐week smartphone application‐based dietary self‐management program was developed through collaboration with a haemodialysis equipment company. The experimental group took this program while the control group took an 8‐week general program. Study variables were serum phosphorus, potassium and albumin, self‐efficacy and quality of life. They were measured at pretest, and 8 weeks and 12 weeks after the programs, and analysed using two‐way repeated measures analysis of variance.

**Findings:**

The smartphone application‐based program significantly improved serum phosphorus, potassium, self‐efficacy and quality of life over time compared with the general program. There was no significant difference in albumin level changes between the groups.

**Conclusions:**

The smartphone application‐based dietary self‐management program is an opportune and effective nursing intervention to lower serum phosphorus and potassium levels in haemodialysis patients over time. Trial registration was performed on www.cris.nih.go.kr (KCT0005366).

**Relevance to clinical practice:**

Haemodialysis patients can easily use the smartphone application to manage their diet anytime and anywhere. They can get real‐time feedback and solutions to prevent haemodialysis complications. Nurses can provide tailored high‐quality care based on an individual's lifelog data from the smartphone application.

**What does this paper contribute to the wider global clinical community?**

- The smartphone application‐based dietary self‐management program developed in this study lowered serum potassium and phosphorus and improved self‐efficacy and quality of life in haemodialysis patients over time.
- Thus, the program assists effective dietary self‐management from the patient's side and aids provision of high‐quality nursing care based on individual lifelog data from the medical team's side, thereby contributing to improving overall health and quality of life of haemodialysis patients.
- The findings also suggest the need to continuously develop various nursing technologies that can be used conveniently by haemodialysis patients in health management.

## 1 INTRODUCTION

The total number of individuals with kidney diseases such as acute kidney injury and chronic kidney disease and those on renal replacement therapy (RRT) exceeds 850 million worldwide (Jager et al., 2019). RRT techniques such as peritoneal dialysis, haemodialysis and kidney transplantation demand lifelong self‐management. The highest proportion of RRT in Korea involves haemodialysis (74.6%), followed by renal transplantation (19.3%) and peritoneal dialysis (6.1%) (Korean End‐Stage Renal Disease [ESRD] Registry Committee, 2019). Haemodialysis replaces about 10% of normal renal function involving aspects such as elimination of water and waste, electrolyte balance and blood pressure control (Korean ESRD Registry Committee, 2019). In general, haemodialysis is carried out three times a week for 3–4 h, and patients require strict diet, exercise and medication management (Niihata et al., 2018); thus, systematic health management of haemodialysis patients is necessary.

If haemodialysis patients do not manage their diet, accumulation of waste products and electrolyte imbalance can lead to serious complications such as oedema, hyponatremia, hyperkalaemia, hypertension, uraemia and eventually death (Niihata et al., 2018). Haemodialysis patients constantly experience the adverse effects of trial‐and‐error approach and inadequate understanding in diet therapy (Lim et al., 2018), and their quality of life often deteriorates owing to the burdens of dietary self‐management (Proscia, 2014).

Most studies of haemodialysis self‐management have used lecture, individual education, video and booklet as education methods (Chan et al., 2019; Frih et al., 2017; Kim et al., 2013, 2015; Park & Kim, 2019; Ramezani et al., 2019), while there are several studies using smartphones for nursing students, nurses and general patients in Korea (Jeon, 2016; Kim & Park, 2019; Lee et al., 2011). As Korea ranks one in the world in smartphone usage rate (Pew Research Center, 2019) and as a smartphone can be used easily anytime and anywhere, it could be an appropriate medium for self‐care management of haemodialysis patients.

A systematic review has suggested that dietary mobile applications for patients with chronic kidney disease may reduce body weight gain, water intake, potassium intake and sodium intake (Campbell & Porter, 2015). Another pilot study using a mobile application for haemodialysis patients reported the application‐based self‐monitoring to be feasible and acceptable (Welch et al., 2013). However, studies using smartphone applications for dietary self‐management in haemodialysis patients are rare.

Therefore, the purpose of this study was (1) to develop a smartphone application‐based dietary self‐management program to help haemodialysis patients to manage their diet conveniently. In this application, whenever the patients select the type and amount of food to eat or they ate, it automatically displays the detailed biochemical values in the food so as to promote dietary self‐management. Unlike lecture training or video watching, the application also provides real‐time feedback or solutions when patients face risky levels of food elements; (2) to examine the effects of the application‐based program on biochemical indicators (serum phosphorus, potassium and albumin), self‐efficacy and quality of life in haemodialysis patients.

## 2 METHODS

### 2.1 Research design

This study used a prospective, single‐blind, randomised, controlled trial with repeated measures in a haemodialysis centre and was registered on www.cris.nih.go.kr (KCT0005366). The study was performed in accordance with guidelines for reporting parallel group randomised trials (Appendix S1).

### 2.2 Research participants

The participants were 75 patients visiting a haemodialysis centre at a tertiary hospital in South Korea. The inclusion criteria were as follows: (1) aged 19 years or older, (2) undergoing haemodialysis three times a week, (3) having an arteriovenous shunt for 5 months or more, (4) having no impairment of hearing, language or cognition and (5) using smartphones.

**FIGURE 1:**
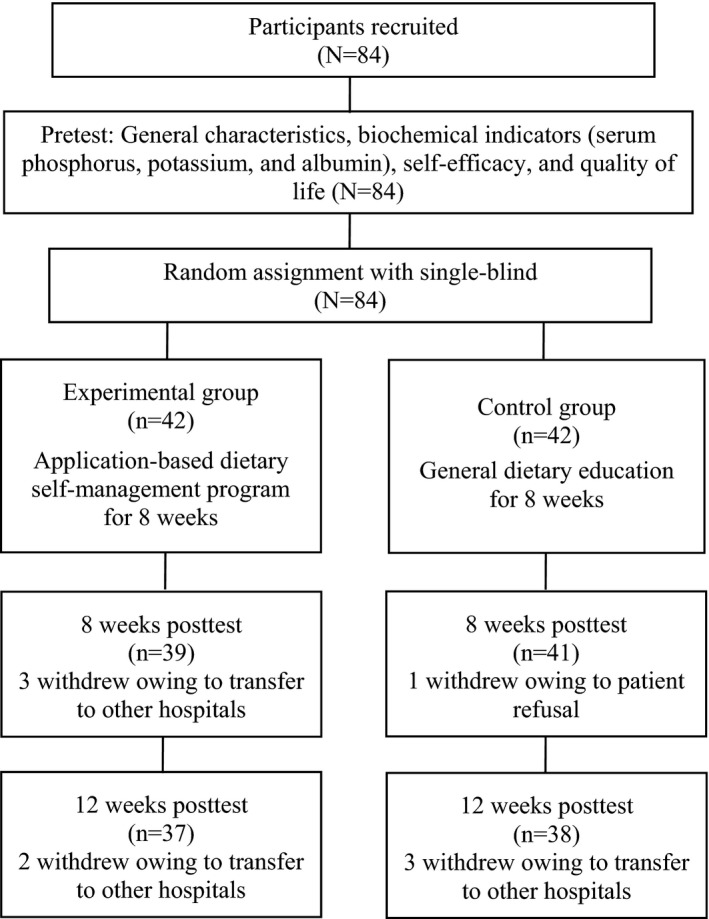
Research process flowchart

The sample size was calculated as 74 participants using G*Power 3.1.9. for the significance level of .05, power of 80% and effect size of .27 for repeated measures analysis of variance (ANOVA) based on a previous study (Kim et al., 2013). Considering the attrition rate, 84 participants were selected for each group. Four participants dropped out during the 8‐week programs and five dropped out at 12 weeks post‐test owing to transfer to other hospitals. Thus, 37 participants from the experimental group and 38 from the control group completed the study (Figure 1).

### 2.3 Intervention

#### 2.3.1 Smartphone application‐based dietary self‐management program group

The goal of the smartphone application‐based dietary self‐management program was to aid dietary management without restrictions of time and space. The application was developed through collaboration with F company with the advice of a haemodialysis expert panel including five haemodialysis nurses, two nephrology physicians and two nursing professors. The contents included introduction screen, diet diary, searching food, favourite food, frequent food, current food, recommended food, my chart and diet therapy (Figure S1). When patients select the type and amount of food they ate or plan to eat, the application screen automatically displays the simulated values of calories, protein, phosphorus, sodium, potassium and albumin in food.

**FIGURE 2:**
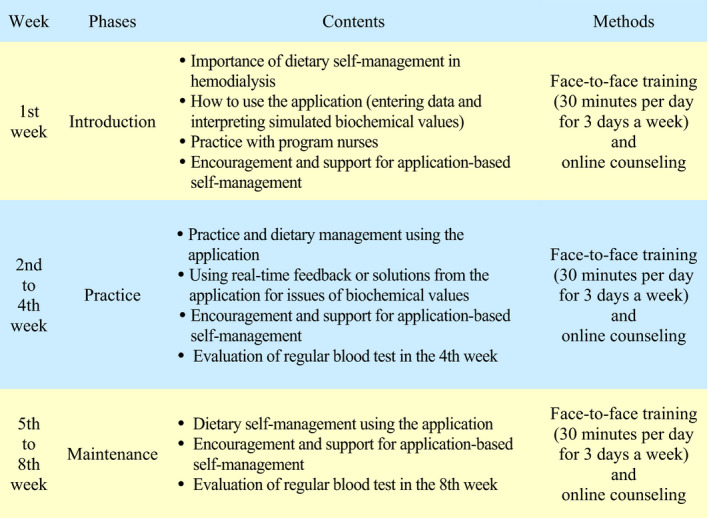
The smartphone application‐based dietary self‐management program

The 8‐week program included introduction, practice and maintenance phases (Figure 2). A 30‐min training session per day was provided for 3 days a week through face‐to‐face training at the haemodialysis centre and online counselling using smartphones while patients were at home. In the introduction phase (first week), patients learned how to download the application to the smartphone, input basic information, use the application to enter meal details and how to recognise the simulated biochemical values and recommended diets, etc., and then they practiced.

In the practice phase (second to fourth week), based on the real‐time feedback of simulated values from the application, participants could recognise risks in health status and manage their diet by themselves. In the fourth week, they took regular blood tests and obtained feedback on diet plan solutions based on the results. In the maintenance phase (fifth to eighth week), participants maintained their diet self‐management using the application and took regular blood tests. In the eighth week, they again obtained feedback on diet plan solutions based on the regular blood test results. The content validity index (CVI) was calculated as .92 from the expert panel.

#### 2.3.2 General dietary program group

The participants in the control group were provided the 8‐week general dietary program for diet self‐management for 3 days a week at the haemodialysis centre through 30‐min face‐to‐face training with booklets. Through this training, the program nurse educated patients on how to manage diet using the phosphorus and potassium food table, fluid intake and low salt diet, interpreted problematic issues in the diet and blood test results, and encouraged dietary self‐management. While patients were at home, online counselling through smartphone was provided. The CVI was calculated from the expert panel as .89.

### 2.4 Measurements

#### 2.4.1 Biochemical indicators

The biochemical indicators were serum phosphorus (mg/ml), serum potassium (mEq/L) and serum albumin (mg/ml). These values were obtained from monthly blood tests at the haemodialysis centre. We analysed the results before beginning the 8‐week dietary self‐management programs (pretest), 8 weeks later and 12 weeks later for the experimental and control groups.

#### 2.4.2 Self‐efficacy

Self‐efficacy was measured using the 15‐item dietary self‐efficacy questionnaire for haemodialysis patients developed by Seo et al. (2012). The tool measures whether patients are confident to carry out dietary guidance in 15 situations such as fluid restriction and lack of support. The items are rated on a five‐point Likert scale. Higher the score, higher the self‐efficacy. In the study by Seo et al. (2012), Cronbach's alpha was .94. In this study, Cronbach's alpha was .92.

#### 2.4.3 Quality of life

Quality of life was measured using the 18‐item Korean version of Kidney Disease Quality of Life Instrument‐Short Form (KDQOL‐SF) developed by Hays et al. (1994) and translated by Park et al. (2007). It consists of (1) burden of kidney disease (four items), (2) difficulties in activity of daily living (eight items), (3) cognitive problems (three items) and social interaction (three items). The items are rated on a five‐point Likert scale. The negative items were inversely coded. Higher the score, higher the quality of life. In a study by Shim (2010) of haemodialysis patients, Cronbach's alpha was .86. In this study, Cronbach's alpha was .91.

### 2.5 Data collection

Study flyers were posted on bulletin boards at the haemodialysis centre. Participants who met the inclusion criteria were recruited and provided with an explanation of the study. All the participants voluntarily submitted written informed consent after obtaining full explanation of the study from the measurement researchers. Participants were from two groups—from the Monday, Wednesday and Friday schedule and from the Tuesday, Thursday and Saturday schedule. The groups were randomly assigned to experimental or control groups by coin flipping, but the participants did not know whether they were in the experimental or control group.

The experimental group followed the 8‐week smartphone application‐based dietary self‐management program while the control group followed the 8‐week general dietary program conducted by two program nurses per group. Pretest and post‐test values were collected by measurement researchers. Post‐test measurements were conducted at 8 and 12 weeks after pretest. Data collection was conducted from 10 January 2017–6 May 2018.

### 2.6 Ethical considerations

The study was approved by the institutional review board at a tertiary hospital (KHUH‐2016‐11‐042‐006). Participants gave informed consent voluntarily. The informed consent forms included human rights of self‐determination, privacy, confidentiality and fair treatment along with the study purpose and process. All data were treated anonymously with study identification number. After completing the study, the control group participants were also provided with the smartphone application‐based dietary self‐management program, if they wanted.

### 2.7 Statistical analysis

The collected data were analysed with IBM SPSS Statistics 24 for Window (IBM Corporation, Armonk, NY, USA). The general characteristics of the participants were analysed through descriptive statistics. The homogeneity of the two groups was analysed using chi‐squared test and independent samples *t* test. The Shapiro–Wilk normality test was performed to test whether the scores of the two groups were normally distributed. The study variables were analysed using two‐way repeated‐measures ANOVA. The validity of the program contents for both groups was measured through CVI. The internal consistency reliability of the instruments was tested using Cronbach's α coefficients.

## 3 RESULTS

### 3.1 General characteristics of the participants

The majority of participants were male (61.3%), and the mean age was 52.00 ± 10.01 for the experimental group and 50.66 ± 9.15 for the control group. There were no significant differences in gender, age, underlying disease, marital status, duration of haemodialysis, religion, employment, type of health insurance and study variables between the two groups (Table 1).

**TABLE 1:** Homogeneity test for general characteristics and study variables (*N* = 75)

Variables	Total (*N* = 75)	Exp. (*n* = 37)	Con. (*n* = 38)	*t/x* ^2^	*p*
	*M* ± *SD*/*n* (%)	*M* ± *SD*/*n* (%)	*M* ± *SD*/*n* (%)		
Gender					
Men	46 (61.3)	22 (59.5)	24 (63.2)	0.11	.463
Women	29 (38.7)	15 (40.5)	14 (36.8)		
Age (years)	51.32 ± 9.55	52.00 ± 10.01	50.66 ± 9.15	0.61	.546
31–40	12 (16.0)	7 (18.9)	5 (13.2)	0.72	.868
41–50	26 (34.6)	12 (32.5)	14 (36.8)		
51–60	22 (29.3)	10 (27.0)	12 (31.6)		
≥61	15 (20.1)	8 (21.6)	7 (18.4)		
Underlying disease					
Hypertension	29 (38.7)	15 (40.5)	14 (36.8)	1.29	.525
Diabetes	32 (42.7)	17 (46.0)	15 (39.5)		
Others	14 (18.6)	5 (13.5)	9 (23.7)		
Marital status					
Married	56 (74.7)	24 (64.9)	32 (84.2)	3.71	.054
Unmarried	19 (25.3)	13 (35.1)	6 (15.8)		
Experience of haemodialysis (years)					
1–2	9 (12.0)	5 (13.5)	4 (10.6)	1.81	.406
3–4	28 (37.3)	11 (29.7)	17 (44.7)		
≥5	38 (50.7)	21 (56.8)	17 (44.7)		
Religion					
Christianity	41 (54.7)	20 (54.1)	21 (57.1)	0.97	.614
Buddhism	14 (18.6)	8 (21.6)	6 (17.9)		
None	20 (26.7)	9 (24.3)	11 (29.0)		
Educational level					
≤Elementary school	6 (8.0)	3 (8.1)	3 (7.9)	4.86	.183
Middle school	22 (29.3)	7 (18.9)	15 (39.5)		
High school	34 (45.4)	21 (56.8)	13 (34.2)		
≥College	13 (17.3)	6 (16.2)	7 (18.4)		
Employment					
None	18 (24.0)	8 (21.6)	10 (26.3)	1.27	.737
Business	31 (41.4)	14 (37.9)	17 (44.7)		
Professionals	13 (17.3)	8 (21.6)	5 (13.2)		
Housekeeping	13 (17.3)	7 (18.9)	6 (15.8)		
Health insurance					
Medical insurance	64 (85.3)	32 (86.5)	32 (84.2)	0.08	.781
Medical aid	11 (14.7)	5 (13.5)	6 (15.8)		
Phosphorus	6.31 ± 1.05	6.34 ± 1.05	6.28 ± 1.07	0.23	.819
Potassium	5.20 ± 0.40	5.21 ± 0.40	5.19 ± 0.41	0.20	.843
Albumin	3.73 ± 0.43	3.74 ± 0.45	3.72 ± 0.42	0.17	.869
Self‐efficacy	47.72 ± 9.74	47.00 ± 9.33	48.42 ± 10.19	−0.63	.531
Quality of life	54.01 ± 10.62	53.22 ± 10.82	54.79 ± 10.50	−0.64	.525

Abbreviations: Con., control group; Exp., experimental group; *M*, mean; *SD*, standard deviation.

### 3.2 Effects of the smartphone application‐based dietary self‐management program

Among the biochemical indicators, serum phosphorus levels of the experimental group decreased from 6.34 at pretest to 5.03 at 8 weeks post‐test and slightly increased to 5.45 at 12 weeks post‐test while those of the control group decreased from 6.28–5.94 and then increased to 6.11. The two‐way repeated‐measures ANOVA found a significant time by group interaction (*F* = 50.31, *p* < .001), an effect of time (*F* = 17.59, *p* < .001) and a statistically significant difference between the two groups (*F* = 4.89, *p* = .030).

Serum potassium levels in the experimental group decreased from 5.21–4.55 at 8 weeks post‐test and slightly increased to 4.86 at 12 weeks post‐test while those in the control group slightly decreased from 5.19–5.03 and then increased to 5.12. The repeated measures analysis showed a significant time by group interaction (*F* = 37.01, *p* < .001), an effect of time (*F* = 101.40, *p* < .001) and a significant difference between the two groups (*F* = 5.66, *p* = .020).

Serum albumin levels in the experimental group slightly increased from 3.74–3.80 at 8 weeks and 3.79 at 12 weeks post‐test while those in the control group changed from 3.72–3.79 and 3.75. The time by group interaction (*F* = 0.28, *p* = .754) and the difference between the two groups (*F* = 0.07, *p* = .788) were not significant.

The self‐efficacy scores of the experimental group increased from 47.00–58.43 and then slightly decreased to 55.14 while those of the control group changed from 48.42–1.68 and 50.00. The repeated measures analysis found a significant time by group interaction (*F* = 49.46, *p* < .001) and an effect of time (*F* = 147.57, *p* < .001), but a nonsignificant difference between the two groups (*F* = 2.23, *p* = .140).

**FIGURE 3:**
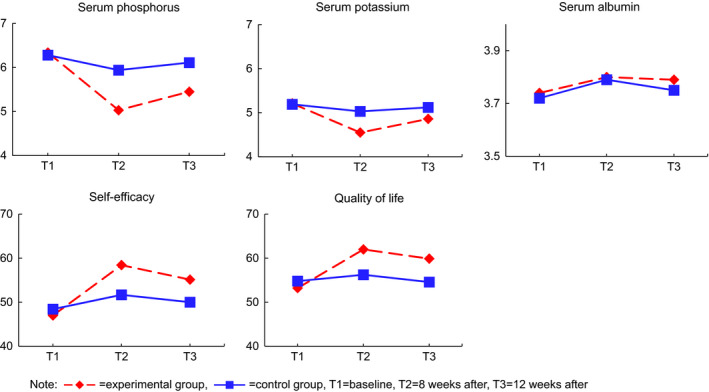
Changes in study variables over time

The quality of life scores of the experimental group increased from 53.22–62.00 and slightly decreased to 59.89 while those of the control group changed from 54.79–56.24 and 54.58. The repeated measures analysis showed a significant time by group interaction (*F* = 56.67, *p* < .001) and an effect of time (*F* = 89.73, *p* < .001), but a nonsignificant difference between the two groups (*F* = 1.65, *p* = .204) (Table 2) (Figure 3).

**TABLE 2:** Changes in biochemical indicators, self‐efficacy and quality of life (*N* = 75)

Variable	Measures	Exp. (*n* = 37)	Cont. (*n* = 38)	Source	*F*	*p*	LSD
		*M* ± *SD*	*M* ± *SD*				
Phosphorus	Baseline	6.34 ± 1.05	6.28 ± 1.07	Group	4.89	.030	B < C < A
	8 weeks	5.03 ± 1.01	5.94 ± 1.03	Time	17.59	<.001	
	12 weeks	5.45 ± 0.97	6.11 ± 1.01	Group * Time	50.31	<.001	
Potassium	Baseline	5.21 ± 0.40	5.19 ± 0.41	Group	5.66	.020	B < C < A
	8 weeks	4.55 ± 0.51	5.03 ± 0.49	Time	101.40	<.001	
	12 weeks	4.86 ± 0.48	5.12 ± 0.46	Group * Time	37.01	<.001	
Albumin	Baseline	3.74 ± 0.45	3.72 ± 0.42	Group	0.07	.788	A, C < B
	8 weeks	3.80 ± 0.42	3.79 ± 0.37	Time	4.44	.013	
	12 weeks	3.79 ± 0.44	3.75 ± 0.37	Group * Time	0.28	.754	
Self‐efficacy	Baseline	47.00 ± 9.33	48.42 ± 10.19	Group	2.23	.140	A < C < B
	8 weeks	58.43 ± 10.80	51.68 ± 10.92	Time	147.57	<.001	
	12 weeks	55.14 ± 10.81	50.00 ± 9.94	Group * Time	49.46	<.001	
Quality of life	Baseline	53.22 ± 10.82	54.79 ± 10.50	Group	1.65	.204	A < C < B
	8 weeks	62.00 ± 12.34	56.24 ± 10.20	Time	89.73	<.001	
	12 weeks	59.89 ± 11.13	54.58 ± 10.08	Group * Time	56.67	<.001	

Abbreviations: A, baseline; B, 8 weeks; C, 12 weeks; Con., control group; Exp., experimental group.

## 4 DISCUSSION

Dietary self‐management is of utmost importance for haemodialysis patients, but it is very difficult to control eating habits for a long time (Gebrie & Ford, 2019). This study investigated the effects of an 8‐week smartphone application‐based dietary self‐management program on biochemical indicators, self‐efficacy and quality of life in haemodialysis patients.

A national report found that 98.7% of Koreans in their 50s and 88.8% in 60s were using smartphones as an important essential medium for communication (95.2%) and information acquisition (94.0%) (No et al., 2019). Among the present study participants, those aged 51 years or older made up 48.6% of the experimental group and 50.0% of the control group, and there were no difficulties in using the application in the experimental group. The study findings suggest that smartphone applications for haemodialysis patients can be actively used as self‐management programs regardless of age. Therefore, rather than fearing that training using smartphone applications would be difficult and involve limitations for older adults, nursing technologies should be positively used.

The 8‐week smartphone application‐based dietary self‐management program was effective in lowering serum phosphorus and potassium in haemodialysis patients, but not in increasing serum albumin. The phosphorous values of the control group decreased from 6.28 mg/dl–5.94 and 6.11 mg/dl, but it was still higher than the normal range of 3.5–5.5 mg/dl (St‐Jules et al., 2018). In contrast, in the experimental group using the smartphone application, the phosphorous levels decreased from 6.34 mg/dl–5.03 and 5.45 mg/dl (within the normal range). The result was consistent with that of a study in which serum phosphorus levels in hyperphosphatemic patients undergoing haemodialysis were reduced through a one‐on‐one nutrition education (Sun et al., 2008).

Serum potassium levels also decreased at 8 weeks and 12 weeks in both groups; the control group levels decreased from 5.19 mEq/L–5.03 and 5.12 mEq/L, but were higher than the normal range of 3.5–5.0 mEq/L (St‐Jules et al., 2018). In the experimental group, the levels decreased from 5.21 mEq/L–4.55 and 4.86 mEq/L (within the normal range). This is consistent with a study of an 8‐week education program that significantly decreased potassium levels in long‐term haemodialysis patients (Kim et al., 2015). Therefore, to continuously manage biochemical indicators such as serum phosphorus and potassium in haemodialysis patients, the application‐based dietary self‐management program may be useful.

The changes in albumin levels of the experimental and control groups did not show a significant difference, which was consistent with the results of a study that showed no effect on albumin after implementing nutrition education and regular exercise program for haemodialysis patients (Kim et al., 2013). No significant changes in albumin levels were found in another study using a multidisciplinary educational approach among haemodialysis patients (Chan et al., 2019) as well as in a randomised controlled trial using interdialytic combined resistance and aerobic exercise training (Frih et al., 2017). Because serum albumin level in haemodialysis patients indicates overall nutritional status and is an important factor in predicting mortality, nutritional supplements may be effective for patients with low serum albumin (Benner et al., 2018). It should be approached with balanced nutrition rather than food intake restrictions. As serum albumin is also an indicator of inflammatory conditions, it is necessary to control associated variables such as inflammation and infection in future studies.

The application‐based dietary self‐management program in this study significantly improved self‐efficacy and quality of life over time among haemodialysis patients. As haemodialysis patients need self‐care activities to cope with their health conditions, nursing interventions that can improve self‐efficacy must be provided. Previous studies on haemodialysis patients also improved patient self‐efficacy through various nursing interventions (Park & Kim, 2019; Ramezani et al., 2019; Yun & Choi, 2016). In the present study, haemodialysis patients could recognise the simulated levels of biochemical indicators in food using real‐time feedback from the application, identify real‐time issues in their eating habits and control their diet, thereby increasing self‐efficacy. Quality of life is very important for patients who need lifelong treatment, such as haemodialysis. Many studies of haemodialysis patients have used exercise programs as interventions to improve quality of life as well as exercise capacity (Huang et al., 2019). Another study involving an 8‐week dietary program also significantly improved quality of life in haemodialysis patients (Yun & Choi, 2016). Therefore, smartphone application‐based dietary self‐management programs can be effective in maintaining a healthy life for long‐term haemodialysis patients by improving self‐efficacy and quality of life.

In this study, the smartphone application‐based dietary self‐management program enabled patients to identify real‐time issues with eating habits and their solutions and to manage diet by themselves. Nurses can also deliver appropriate care considering individual characteristics based on dietary records of such applications. Use of smartphone application is a new and effective approach to the educational system for haemodialysis patients, as it makes it easy to manage diet anytime and anywhere.

## 5 LIMITATIONS

Despite its strengths, this study has some limitations. First, as this study was conducted at a tertiary hospital, to the findings may have limited generalisability. Further studies need to include a larger sample size and more hospital sites. Second, as the program of this study was developed specifically for Koreans, it does not cover diverse food items reflecting various ethnic groups. However, the contents of the program have been revised to include a wide range of ingredients and we continue to expand the range of food. Third, the program of this study focused on nutrition management because it was developed mainly for dietary self‐management. If the smartphone application includes comprehensive management contents including exercise and emotion in addition to diet, it will contribute to the holistic care of haemodialysis patients.

## 6 CONCLUSION

The smartphone application‐based dietary self‐management program lowered serum potassium and phosphorus and improved self‐efficacy and quality of life in haemodialysis patients over time. That is, the program aids effective dietary self‐management from the patient's side and aids provision of high‐quality nursing care based on individual lifelog data from the medical team's side, which will contribute to improving overall health and quality of life of haemodialysis patients. Therefore, it is recommended that haemodialysis patients use the smartphone application‐based dietary self‐management program to form healthy eating habits by themselves. The study findings also suggest that it is necessary to continuously develop various nursing technologies, such as those involving artificial intelligence, to develop applications that are easy and convenient for use among haemodialysis patients.

## 7 CLINICAL RESOURCES

- Korean ESRD Registry Committee. Current renal replacement therapy in Korea. http://www.ksn.or.kr/rang_board/list.html?code=sinchart_eng
- National Kidney Foundation. Home haemodialysis. https://www.kidney.org/atoz/content/homehemo
- Pew Research Center. Smartphone ownership in advanced economies higher than in emerging. https://www.pewresearch.org/global/2019/02/05/smartphone‐ownership‐is‐growing‐rapidly‐around‐the‐world‐but‐not‐always‐equally/pg_global‐technology‐use‐2018_2019‐02‐05_0‐01/

## CONFLICT OF INTEREST

There is no conflict of interest.

## AUTHOR CONTRIBUTIONS

Project conception, collaborative network with university scholars and manuscript writing: J. Lee. Program and synthesisation of results, analysis and conclusions: S. Pack.

## ETHICAL APPROVAL

The study was conducted after the institutional review board (IRB) approval (KHUH‐2016‐11‐042‐006).

## Supporting information

Appendix S1

Fig S1

## Data Availability

The data that support the findings of this study are available from the corresponding author upon reasonable request.
