# Supplementary material for: Randomised controlled trial of a smartphone application‐based dietary self‐management program on haemodialysis patients
Source: J Clin Nurs. 2021 Jan 28;30(5-6):840–8. doi: 10.1111/jocn.15627 (PMC8048988; doi:10.1111/jocn.15627)
Supplement: Supplementary file 2 — Fig S1 [file JOCN-30-840-s002.docx]

| Introduction  screen | 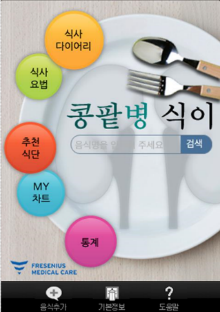 | Introduction screen includes the icons of diet diary, diet therapy, recommended diet, my chart, and statistics. |
| --- | --- | --- |
| Diet diary | 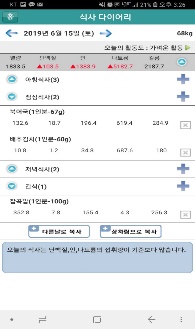 | If you enter the type and amount of food eaten or those which you plan to eat, you will be informed in real time about the simulated values of calories, protein, phosphorus, sodium, and potassium. |
| Searching food | 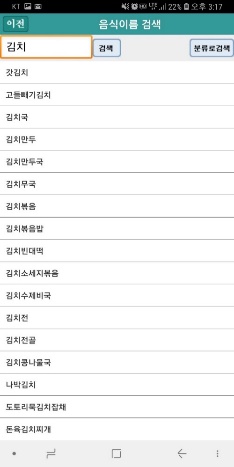 | You can search food by entering the name or selecting from the food list |
| Favorite food | 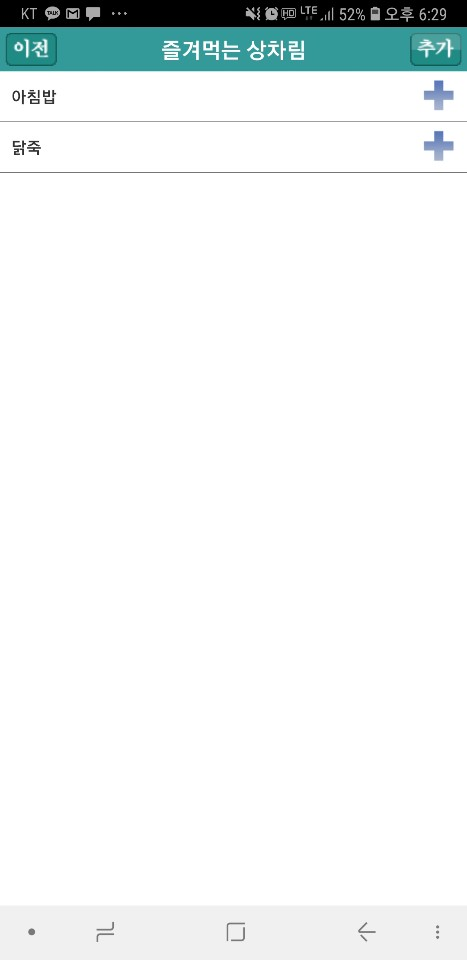 | You can simply add your favorite food to the food list |
| Frequent food | 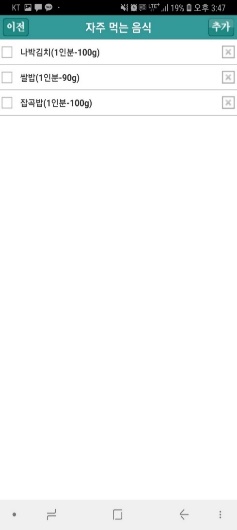 | You can simply select from your frequent food list |
| Current food | 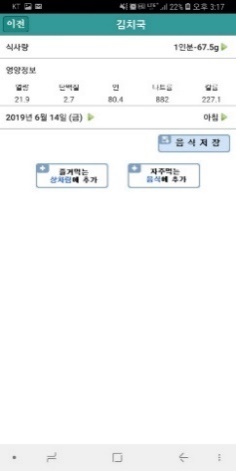 | You can check your current food and its simulated biochemical values |
| Recommended food | 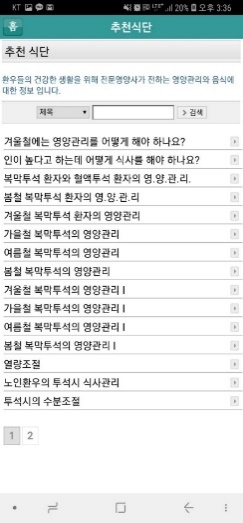 | You can identify recommended food for your conditions |
| My chart | 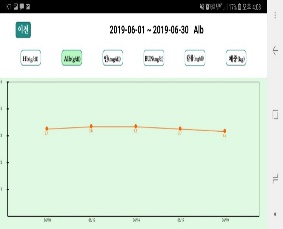 | You can check the daily graph of each biochemical value expected to be accumulated in your body. |
| Diet therapy | 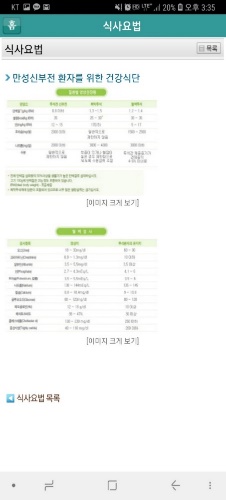 | You can find the dietary guidelines for diet therapy |

Figure S1. The smartphone application for hemodialysis patients
